# Genome-wide association study in patients with posterior urethral valves

**DOI:** 10.3389/fped.2022.988374

**Published:** 2022-09-27

**Authors:** Loes F. M. van der Zanden, Carlo Maj, Oleg Borisov, Iris A. L. M. van Rooij, Josine S. L. T. Quaedackers, Martijn Steffens, Luca Schierbaum, Sophia Schneider, Lea Waffenschmidt, Lambertus A. L. M. Kiemeney, Liesbeth L. L. de Wall, Stefanie Heilmann, Aybike Hofmann, Jan Gehlen, Johannes Schumacher, Maria Szczepanska, Katarzyna Taranta-Janusz, Pawel Kroll, Grazyna Krzemien, Agnieszka Szmigielska, Michiel F. Schreuder, Stefanie Weber, Marcin Zaniew, Nel Roeleveld, Heiko Reutter, Wout F. J. Feitz, Alina C. Hilger

**Affiliations:** ^1^Department for Health Evidence, Radboud Institute for Health Sciences, Radboud University Medical Center, Nijmegen, Netherlands; ^2^Institute for Genomic Statistics and Bioinformatics, Medical Faculty, University of Bonn, Bonn, Germany; ^3^Department of Urology, University Medical Center Groningen, Groningen, Netherlands; ^4^Department of Urology, Isala, Zwolle, Netherlands; ^5^Institute of Human Genetics, School of Medicine and University Hospital Bonn, University of Bonn, Bonn, Germany; ^6^Division of Pediatric Urology, Department of Urology, Radboud Institute for Molecular Life Sciences, Radboudumc Amalia Children's Hospital, Nijmegen, Netherlands; ^7^Department of Pediatric Urology, Clinic St. Hedwig, University Medical Center Regensburg, Regensburg, Germany; ^8^Center for Human Genetics, University Hospital of Marburg, Marburg, Germany; ^9^Department of Pediatrics, Faculty of Medical Sciences in Zabrze, Medical University of Silesia in Katowice, Zabrze, Poland; ^10^Department of Pediatrics and Nephrology, Medical University of Białystok, Białystok, Poland; ^11^Neurourology Unit, Pediatric Surgery and Urology Clinic, Poznań, Poland; ^12^Department of Pediatrics and Nephrology, Medical University of Warsaw, Warsaw, Poland; ^13^Department of Pediatric Nephrology, Radboud Institute for Molecular Life Sciences, Radboudumc Amalia Children's Hospital, Nijmegen, Netherlands; ^14^University Children Hospital Marburg, Philipps University Marburg, Marburg, Germany; ^15^Department of Pediatrics, University of Zielona Góra, Zielona Góra, Poland; ^16^Division of Neonatology and Pediatric Intensive Care, Department of Pediatrics and Adolescent Medicine, Friedrich-Alexander University of Erlangen-Nürnberg, Erlangen, Germany; ^17^Department of Pediatrics and Adolescent Medicine, Friedrich-Alexander University of Erlangen-Nürnberg, Erlangen, Germany; ^18^Research Center on Rare Kidney Diseases, University Hospital Erlangen, Erlangen, Germany

**Keywords:** genome wide association study, lower urinary tract obstruction, obstructive uropathy, posterior urethral valves, *PCDH9*, *SALL1*, *BMP7*

## Abstract

Congenital lower urinary tract obstructions (LUTO) are most often caused by posterior urethral valves (PUV), a male limited anatomical obstruction of the urethra affecting 1 in 4,000 male live births. Little is known about the genetic background of PUV. Here, we report the largest genome-wide association study (GWAS) for PUV in 4 cohorts of patients and controls. The final meta-analysis included 756 patients and 4,823 ethnicity matched controls and comprised 5,754,208 variants that were genotyped or imputed and passed quality control in all 4 cohorts. No genome-wide significant locus was identified, but 33 variants showed suggestive significance (*P* < 1 × 10^−5^). When considering only loci with multiple variants residing within < 10 kB of each other showing suggestive significance and with the same effect direction in all 4 cohorts, 3 loci comprising a total of 9 variants remained. These loci resided on chromosomes 13, 16, and 20. The present GWAS and meta-analysis is the largest genetic study on PUV performed to date. The fact that no genome-wide significant locus was identified, can be explained by lack of power or may indicate that common variants do not play a major role in the etiology of PUV. Nevertheless, future studies are warranted to replicate and validate the 3 loci that yielded suggestive associations.

## Introduction

Congenital lower urinary tract obstruction (LUTO, MIM # 618612) is defined by a decrease in the free passage of urine from the bladder through the urethra. LUTO can be one of the most severe diagnoses underlying obstructive uropathy and may perturb kidney development. Obstructive uropathy is the second most prevalent cause of end-stage kidney disease in children (1). LUTO is most often caused by an anatomical blockage, commonest by posterior urethral valves (PUV) (2), a male limited phenotype affecting 1 in 4,000 male live births (3). Severe forms of LUTO can be diagnosed intrauterine, presenting with megacystis, including “keyhole sign”, as well as megaureter, oligohydramnios, and often dysplastic kidneys (2). Milder LUTO forms may manifest postnatally, often with recurrent urinary tract infections (3). Little is known about the genetic background of anatomical LUTO or PUV, which occur sporadically in most cases. Familial forms, segregating through generations with mixed phenotypes (PUV and stenosis) as well as affected sib-pairs, have been described, however, suggesting a potential genetic contribution to the malformation (4, 5). Furthermore, Frese et al. (6) found higher concordance rates among monozygotic compared to dizygotic twin pairs in a classic twin study. Two genetic studies found rare copy number variants (CNVs) potentially contributing to PUV in up to 57% of cases (7) and a higher occurrence of rare CNVs among PUV patients than among controls (8). In addition, an association with a variant in the angiotensin II receptor type 2 (*AGTR2*) was found in one study (9). Variants in basonuclin 2 (*BNC2*) as a possible first identified monogenic cause were described by Kolvenbach et al. (10). Overall, the genetic background appears heterogeneous with a multifactorial mode of inheritance likely to underlie a large proportion of patients. Here, we report the largest study using genome-wide association methods to identify genetic susceptibility loci for PUV. The final meta analyses comprised a total of 756 patients and 4,823 controls.

## Materials and methods

### Patients and controls and genotyping

Details of the recruitment process for patients and controls are provided in the Supplement. In short, the GWAS sample comprised 823 male PUV patients and 5,254 male controls. This sample consisted of in total 4 cohorts from The Netherlands, Germany, and Poland. Prior to inclusion, written informed consent was obtained from all subjects or from their proxies in case of legal minors. The study was approved by the institutional medical-ethics committee of the participating centers and was conducted in accordance with the principles of the Declaration of Helsinki. DNA was extracted from blood and saliva samples using standard procedures.

All Dutch samples were genotyped by deCODE Genetics (Reykjavik, Iceland). The first Dutch cohort comprised 417 patients derived from the Radboudumc AGORA data- and biobank (11, 12), which were patients born in 1981 or later who underwent a valve resection before the age of 18 years, and 2,493 controls derived from the Nijmegen Biomedical Study, a population-based study in which randomly selected inhabitants of the municipality of Nijmegen received an invitation to donate blood samples (13). This cohort was genotyped using the Infinium OmniExpress bead chips (Illumina, San Diego, CA, USA) and will further be called the Dutch Omni sample. The second Dutch cohort consisted of 62 AGORA patients and 323 controls collected in AGORA by asking 42 municipalities to provide a random sample of their inhabitants in the age range of 0–20 years and inviting these children and adolescents to provide a saliva sample. This cohort was genotyped using the Global Screening Array (GSA) from Illumina modified by deCODE (Illumina, San Diego, CA, USA), and will further be called the Dutch GSA cohort. The German cohort comprised 109 diagnosed PUV patients sampled through the CaRE for LUTO (Cause and Risk Evaluation for LUTO) study, mostly included within in the first year of life right after diagnosis of PUV and 2,144 German controls derived from a prospective population-based cohort study, the Heinz Nixdorf Recall Study (14). The Polish sample comprised 235 diagnosed PUV patients recruited in a collaboration between the CaRE for LUTO Study and the PolTubeReg (Polish registry for tubular kidney disease) and 294 Polish controls, which were recruited as blood donors in Lodz, Poland. The German and Polish patient and control samples were genotyped using the Global Screening Array I (Illumina, San Diego, CA, USA).

### Quality control and imputation

#### Pre-imputation quality control

Quality control was performed separately for samples from the different populations and for samples genotyped on different platforms. Variants were excluded if they had < 98% call rate, had minor allele frequencies (MAF) < 0.1%, or failed the Hardy Weinberg Equilibrium (HWE) test (*P* < 1 × 10^−10^ for patients or *P* < 1 × 10^−6^ for controls). Samples were excluded if they had discordant sex information or a call rate < 98%, or if the kinship coefficient calculated using KING (15) was >0.0442, indicating a 3rd-degree relationship or higher with another sample in the cohort. In the identification of individuals from other ethnicities, PLINK was used to compute principal components (PC) for study participants and reference samples of the 1,000 Genomes Project Phase 3 (16) and to visualize sub-structuring. Participants that clustered with a non-European population from the 1,000 Genomes project were excluded. We recomputed the PC in the remaining participants only, visualized results, and excluded participants with the first or second PC more than 6 standard deviations from the mean.

#### Imputation

Imputation was performed for hg19 built, separately for samples from the different populations and for samples genotyped on different platforms. Genotypes of the different samples were phased using SHAPEIT (v2) (17) and imputed using IMPUTE2 (v2) (18) using 1,000 Genomes Project Phase 3 data as a reference panel (16). After imputation, variants with an IMPUTE2 information metric (info) score < 0.6 or with a minor allele count < 20 were excluded.

### Association analysis

Association analysis was performed separately for samples from the different populations and for samples genotyped on different platforms in PLINK using the logistic regression framework implemented in the glm function of PLINK 2,^1^ adjusting for the first 4 principal components. Afterwards, results of SNPs that were genotyped or imputed and passed quality control in all cohorts were pooled in a meta-analysis using the effect size estimates and standard errors in the METAL software, SCHEME STDERR.^2^ The qqman package in R^3^ was used to visualize the results by means of Manhattan and Q-Q plots. The metafor package in R^4^ was used to create forest plots for selected genetic variants of interest.

## Results

The post-quality control dataset forwarded to independent GWAS comprised 402 patients and 2,213 controls for the Dutch Omni cohort, 58 patients and 314 controls for the Dutch GSA cohort, 104 patients and 2,070 controls for the German cohort, and 192 patients and 226 controls for the Polish cohort. The independent GWAS in the German cohort reached genome-wide significance for 3 genotyped variants on chromosome 16 (rs117706747, rs117689220, and rs17666927). Imputation yielded 75 additional variants in LD that also showed genome-wide significance (ORs ranging between 5.65 and 8.52 with *P*-values of 5E^−8^ to 8E^−11^). The results in the Polish and the Dutch GSA cohorts showed the same effect direction as in the German cohort (ORs ranging between 1.28 and 2.14 with *P*-values of 0.51–0.02 for the Polish cohort and OR between 1.65 and 1.70 with *P*-values of 0.14–0.09 for the Dutch GSA cohort), whereas the Dutch Omni cohort presented with opposite or no effect directions (ORs ranging between 0.66 and 1.18 with all *P*-values > 0.11) (Supplementary Table 1).

**Figure 1:**
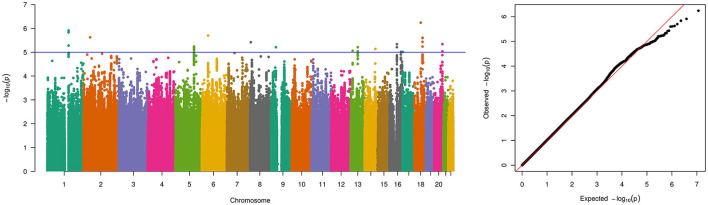
Manhattan and quantile-quantile (QQ)-plot of pooled genome-wide association study results for the 5,754,208 variants that were genotyped and passed quality control in all 4 PUV cohorts for a total number of 756 patients and 4,823 controls. The blue line indicates the threshold for suggestive genome-wide significance (*p*-value of 1 × 10^−5^).

**Figure 2:**
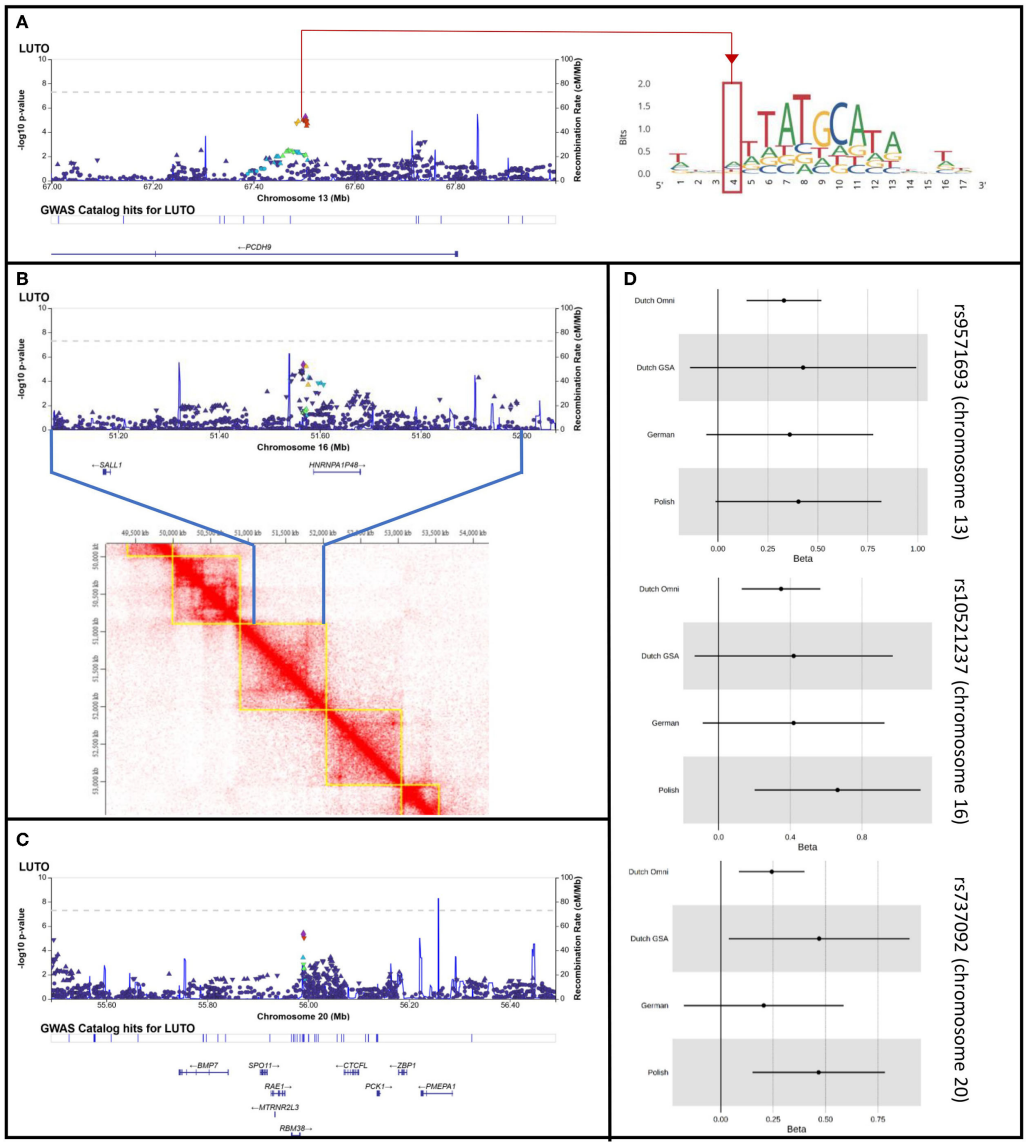
LocusZoom plots for the loci on chromosome 13, 16, and 20 with multiple variants residing within < 10 kB of each other showing suggestive significance (*P* < 1 × 10^−5^) and with the same effect direction in all 4 cohorts. **(A)** Locus Zoom plot for chr. 13, with LD scoring referring to top SNP rs9571693. Red line and red quarter indicate rs34844007 in locus zoom plot and in position weight matrix ENCFF525TXJ targeted by POU class family genes. **(B)** Locus Zoom plot for chr. 16, with LD scoring referring to top SNP rs10521237. Heat map visualization of Hi-C interactions among parts of Chr.16q12.1 and parts of 16q12.2 from HMEC human kidney cells, as plotted by TADKB. Blue lines indicate the region plotted in the locus zoom, showing the identified locus and *SALL1* to locate in the same topological associated domain. **(C)** Locus Zoom plot for chr. 20, with LD scoring referring to top SNP rs737092. **(D)** Forest plots for rs9571693, rs10521237, and rs737092, the leading SNPs of the loci on chromosome 13, 16, and 20, respectively.

The final meta-analysis included 756 patients and 4,823 on ethnicity matched controls and comprised 5,754,208 variants that were genotyped or imputed and passed quality control in all 4 cohorts. No relevant inflation was detected (λ = 1.01) and no genome-wide significant locus was identified (Figure 1). In total, 33 variants showed suggestive significance (*P* < 1 × 10^−5^, see Supplementary Table 2). However, the effects were in different directions across cohorts for some of the loci (i.e., on chromosome 1, 5, and 18). When considering loci with multiple variants residing within < 10 kB of each other showing suggestive significance and with the same effect direction in all 4 cohorts only, 3 loci comprising a total of 9 variants remained (Table 1). These loci reside on chromosome 13 (5 variants), 16 (2 variants), and 20 (2 variants) (see Figures 2A–C for LocusZoom plots and Figure 2D for Forest plots of leading variants at these loci). While the locus on chromosome 13 resides in an intronic region of the gene *PCDH9*, the loci on chromosome 16 and 20 reside in intergenic regions.

**Table 1:** Association results with PUV for the variants in loci with multiple variants residing within < 10 kB of each other showing suggestive significance (*P* < 1 × 10^−5^) and with the same effect direction in all 4 cohorts.

**CHR**	**BP**	**dbSNPrs#**	**A1**	**Meta analysis results**			**Association results in the 4 cohorts**							
							**Dutch GSA**		**Dutch Omni**		**German**		**Polish**	
				**P_**het**_**	**OR**	**P_**OR**_**	**OR_**a**_**	**P_**a**_**	**OR_**a**_**	**P_**a**_**	**OR_**a**_**	**P_**a**_**	**OR_**a**_**	**P_**a**_**
13	67498549	rs9564361	A	0.97	1.40	9 × 10^−6^	1.58	0.10	1.39	4 × 10^−4^	1.34	0.15	1.41	0.09
13	67498767	rs34844007	C	0.98	1.40	1 × 10^−5^	1.57	0.11	1.39	4 × 10^−4^	1.34	0.15	1.41	0.10
13	67499094	rs34948729	A	0.98	1.40	1 × 10^−5^	1.57	0.11	1.39	4 × 10^−4^	1.34	0.15	1.41	0.10
13	67502603	rs9571693	G	0.98	1.42	6 × 10^−6^	1.53	0.14	1.39	5 × 10^−4^	1.43	0.09	1.50	0.06
13	67503594	rs9571694	A	0.99	1.41	1 × 10^−5^	1.45	0.20	1.39	7 × 10^−4^	1.47	0.07	1.46	0.08
16	51567231	rs10521237	G	0.69	1.51	5 × 10^−6^	1.52	0.14	1.42	2 × 10^−3^	1.52	0.11	1.94	5 × 10^−3^
16	51572611	rs67018781	T	0.83	1.50	6 × 10^−6^	1.53	0.13	1.44	1 × 10^−3^	1.43	0.16	1.82	0.01
20	55990405	rs737092	C	0.49	1.34	5 × 10^−6^	1.60	0.03	1.28	2 × 10^−3^	1.23	0.29	1.60	4 × 10^−3^
20	55991637	rs6014993	G	0.59	1.32	9 × 10^−6^	1.59	0.03	1.27	3 × 10^−3^	1.20	0.34	1.50	8 × 10^−3^

CHR, chromosome; BP, basepair; dbSNPrs#, rs-number as provided by dbSNP; A1, effect allele; P_het_, P-value indicating whether statistical significant heterogeneity was present among the 4 cohorts; OR, odds ratio; P_OR_, P-value for odds ratio; OR_a_, odds ratio adjusted for the first principal component; P_a_, P-value for adjusted odds ratio; Dutch GSA, Dutch cohort genotyped on the Global Screening arrays; Dutch Omni, Dutch cohort genotyped on the Human OmniExpress BeadChips; German, German cohort; Polish, Polish cohort.

## Discussion

Here, we report the first GWAS aiming to identify susceptibility loci in patients with PUV. GWASs in 4 different cohorts revealed one genome-wide significant locus in the German cohort on chromosome 16. However, the strong signal could not be replicated in the other cohorts (see Supplementary Figures 1–4; Supplementary Table 1). Although not genome-wide statistically significant, the cohorts genotyped on Global Screening Arrays (the German, Polish, and Dutch GSA cohorts) all show the same direction of effect, whereas the largest cohort in this study, the Dutch Omni cohort, showed the opposite effect direction. Whether this array-specific correlation might be caused by a genotyping effect or is due to the different sample sizes remains speculative. Still, a batch effect driving the very strong German signal seems unlikely as the German and Polish patients were processed at the same time, physically sharing the same genotyping chips. Nevertheless, with one cohort showing an opposite effect direction, we consider this locus not to be a PUV-risk locus, albeit its strong positive signal in the German cohort.

In the meta-analysis of the 4 GWASs, no genome-wide significant association was identified. We would like to emphasize that we used very conservative selection criteria only including variants that passed quality control in all 4 cohorts. While the usage of multiple cohorts and thus the exclusion of many variants might be one limitation of this study, it also guaranteed that only high-quality data were used in the final analysis, excluding the possibility that batch effects or signals driven by a single cohort could lead to false positive findings in the meta-analysis. Hence, technical issues that can occur by using just a single genotyping platform could be excluded by the usage of multiple genotyping platforms. The fact that we did not observe genome-wide significant results may indicate that common variants do not play a major role in the etiology of PUV. Of note, by excluding variants with an allele count < 20 in the single cohorts, rare variants were excluded because of the low number of patients and controls, especially in the Dutch GSA (*N* = 372 individuals) and the Polish (*N* = 418 individuals) cohorts. Our lack of statistically significant results could also be explained by the relatively small number of patients and the resulting lack of power, which is a further limitation of this study and caused by the fact that PUV are a rare disease. With 756 patients and 4,823 controls in a single cohort, we would have had 80% power to detect loci with an allele frequency of 0.10 and an OR of 1.3. ORs of this magnitude are generally not observed for multifactorial disorders, although previous GWASs of similar size for other rare birth defects such as hypospadias, bladder extrophy and cleft lip and palate were able to identify loci of genome-wide significance (19–21).

We identified 3 loci with multiple variants showing suggestive significance (*P* < 1 × 10^−5^) and with the same effect direction in all 4 cohorts. We will discuss these loci here in the realization that our results are mainly to be regarded as suggestive evidence for association and that larger studies are needed to confirm our findings. In the first locus on chromosome 13, 5 variants reached *P*-values below 1 × 10^−5^. The other loci, located on chromosomes 16 and 20 (Table 1), harbored 2 variants each with a *P*-value below 1 × 10^−5^.

### Chromosome 13

The signal on chromosome 13 is located within the first intron of the *PCDH9* gene (Figure 2A). Previous studies showed associations with body mass index, adult body size, and urinary potassium excretion (GWAS catalog)^5^ None of these phenotypes can be linked directly with PUV. The *PCDH9* gene encodes a member of the protocadherin family, a group of proteins that mediate cell adhesion in neural tissues in the presence of calcium (22). PCDH9 may be involved in signaling at neuronal synaptic junctions. Hence, expression in adult tissues has been found strongest in the brain, while weak expression was found in nearly all organs including the urinary tract and prostate^6^. In the EMBL-EBI expression atlas (23), experiment # E-MTAB-6592 shows constant expression of *PCDH9* in developing human urinary bladder and genital tissues through gestational weeks 7–9. Approximately one third of genes that were found to be expressed in this experiment, however, showed higher expression then *PCDH9*. Still, PCDH9 might be involved in urinary tract development.

Direct functional importance of the locus is predicted in the Regulome Database^7^ (24), which gives regulatory probability scores ranging from 0 to 1, with 1 being most likely to be a regulatory variant, generated using a machine learning approach. Rs34844007, one of the 5 associated variants, has a probability score of 1 and is therefore very likely to be a regulatory variant. This variant is located within a transcription factor binding motif which is targeted by 16 members of the POU gene family (Figure 2A). Genes of this family have been shown to be crucially involved in the regulation of developmental processes in many tissues and organs (25). For example, Rieger et al. (26) showed in mice that a missense mutation in *Pou3f* , one of the genes predicted to target the transcription factor binding motif, reduces nephron numbers and impairs development of the thick ascending limb of the Loop of Henle. Nevertheless, the chromatin state in all investigated embryonic cell lines in the Regulome DB is indicative of low or quiescent transcription. Thus, association of the specific transcription factor binding motif with the development of the lower urinary tract awaits further evaluation.

### Chromosome 16

The 2 variants with suggestive significance located on chromosome 16 are located within an intergenic region (Figure 2B). Located 40 kb upstream from our top variants, the GWAS catalog lists a genome-wide significant association with the use of diuretics and the use of agents acting on the renin-angiotensin system (top variant rs62039768, *p*-value 1 × 10^−8^, location chr16:51526850) (27), but no functional annotation was given in this publication. Both medications are frequently used in patients with chronic kidney disease, which is often seen in PUV patients. The closest protein coding gene, located 14kb downstream of our top locus, is *HNRNPA1P48*. This gene is a pseudogene to *HNRPA1*, variants of which have been implicated in amyothrophic lateral sclerosis, a phenotype not associated with LUTO (28). The pseudogene is likely to be expressed in the nucleus (www.proteinatlas.org) and little is known about its functional implications. The locus is flanked by a second gene, *SALL1*, located 387 kb upstream the lead SNP at this locus. According to existing data of topological associated domains (TAD) within several human adult and embryonic cell types (HMEC, NHEK, IMR-90, HUVEC, data source)^8^, our top locus locates in one topologically associated domain with *SALL1* (Figure 2B). This is particularly interesting as autosomal dominantly inherited variants in *SALL1* are known to cause Townes-Brocks syndrome (MIM# 107480), with genitourinary malformations being one of the syndromes features (29). Especially as PUV have been reported in single cases of Townes-Brocks syndrome (30, 31). Functional studies could shed more light on this correlation.

### Chromosome 20

The signal on chromosome 20 is also located within an intergenic region (Figure 2C). Both variants reaching suggestive significance in this locus have been described in several GWASs and a meta-analysis focusing on erythrocyte traits and erythropoiesis (32). In these studies, no direct functional annotation was provided.

Several genes lie in close proximity to this locus, with *RBM38* being the closest. In mice this gene is described to be weakly expressed during development of genitourinary system, but KO-mice do not show any genitourinary phenotype.^9^ Interestingly, *BMP7*, which locates within 150 kb distance, is described to be expressed in the developing murine urinary tract. Loss of *Bmp7* has been shown to result in arrest of cloacal septation and in severe abnormalities of the morphogenesis of the genital urethra and mesenchyme (33). Furthermore, heterozygous deletion of this region in human, including the here identified risk locus of suggestive significance, was described in a patient with anatomical LUTO and in one patient from Decipher with hydronephrosis (7). As these are only single case descriptions, larger CNV studies on LUTO patients need to prove that heterozygous loss of the region can be causal for the phenotype.

## Conclusion

The present GWAS and meta-analysis is the largest genetic study on PUV performed to date. The fact that no genome-wide significant loci could be identified, may indicate that common variants do not play a major role in the etiology of PUV or could be explained by a lack of power. Nevertheless, 3 loci yielded suggestive associations. Future studies are warranted to support these loci. Ideally, by confirming them either, in a replication study of a single very large cohort or by adding more cohorts to the performed meta-analysis to reach genomewide significance. Nevertheless, considering PUV being a rare disease sampling such a cohort will take some time. Nevertheless, in the meantime, loci could be further explored by performing sequencing of the complete locus regions in cases and controls to identify variants in exonic, intronic, or extragenic regions that are overrepresented in cases compared to controls, suggesting them to be implicated in the phenotype.

## Data availability statement

The data presented in the study are deposited in the GWAS Catalog (https://www.ebi.ac.uk/gwas/), accession number GCP000376.

## Ethics statement

The Arnhem-Nijmegen Regional Committee on Research Involving Human Subjects approved the AGORA project, the Nijmegen Biomedical Study, and the current study. The board of directors of the Isala clinics and UMCG approved implementation of AGORA in these centers. The German and Polish studies involving human participants were reviewed and approved by Ethikkommission der Friedrich-Alexander-Universität Erlangen-Nürnberg, Krankenhausstraße 12, 91054 Erlangen. Written informed consent to participate in this study was provided by the participants' legal guardian/next of kin.

## Author contributions

CM and OB performed GWAS analysis. LS, SS, LdW, SH, JG, JS, and ACH performed genotyping of different patient and control cohorts. LvZ, IvR, JQ, MSt, LK, LdW, AH, JS, MSz, KT-J, PK, GK, AS, MFS, SW, MZ, NR, HR, WF, and ACH recruited families for the study. LvZ and ACH designed and oversaw the entire study and wrote the manuscript together with CM and HR. All authors approved and commented the manuscript.

## Funding

This work was supported by a Kolff junior postdoc grant from the Dutch Kidney Foundation (13OKJ36) and a ZonMW-VENI grant from the Netherlands Organization for Scientific Research (91618036) to LvZ, by the German Research Foundation (DFG, RE 1723/4-1) and by the Else-Kröner-Fresenius-Stiftung (EKFS, 2014_A14) to HR and by BONFOR grant O-149.0123, and the Else Kröner-Fresenius-Stiftung and the Eva Luise und Horst Köhler Stiftung—Project No: 2019_KollegSE.04 to ACH. We acknowledge financial support by Deutsche Forschungsgemeinschaft and Friedrich-Alexander-Universität Erlangen-Nürnberg within the funding program “Open Access Publication Funding.”

## Conflict of interest

Author SH was employed by Life&Brain GmbH.

The remaining authors declare that the research was conducted in the absence of any commercial or financial relationships that could be construed as a potential conflict of interest.

## Publisher's note

All claims expressed in this article are solely those of the authors and do not necessarily represent those of their affiliated organizations, or those of the publisher, the editors and the reviewers. Any product that may be evaluated in this article, or claim that may be made by its manufacturer, is not guaranteed or endorsed by the publisher. Provide the complete details for the following references.

## Footnotes

^1^ https://www.cog-genomics.org/plink/2.0/

^2^ https://genome.sph.umich.edu/wiki/METAL

^3^ https://www.r-project.org/nosvn/pandoc/qqman.html

^4^ http://www.metafor-project.org/doku.php

^5^ https://www.ebi.ac.uk/gwas/

^6^http://www.proteinatlas.org version 20.1.

^7^ https://regulomedb.org

^8^http://dna.cs.miami.edu/TADKB/)

^9^http://www.informatics.jax.org/ version MGI 6.21.
