# Supplementary material for: Genome-wide association study in patients with posterior urethral valves
Source: Front Pediatr. 2022 Sep 27;10:988374. doi: 10.3389/fped.2022.988374 (PMC9552614; doi:10.3389/fped.2022.988374)
Supplement: Supplementary file 3 [file Data_Sheet_1.docx]

Supplementary Material

# Patients and controls

## Dutch cohort

AGORA (Aetiologic research into Genetic and Occupational/environmental Risk factors for Anomalies in children) is a data- and biobank in the Radboud university medical center, Nijmegen, the Netherlands, in which questionnaire data, blood/saliva samples and phenotypic information are collected from patients with congenital malformations or childhood cancer and their parents [van Rooij et al. 2016; Manders et al. 2018]. Patients with PUV treated in the Isala clinics in Zwolle and the University Medical Center in Groningen (UMCG) were also asked to participate in AGORA. For the current study, we identified all patients born in 1981 or later who underwent a valve resection before the age of 18 years from the AGORA data- and biobank. In 2010–2011, AGORA controls were recruited by asking 42 municipalities to provide a random sample of their inhabitants in the age range of 0 to 20 years. For the current study, we used the male AGORA controls as well as male controls from the Nijmegen Biomedical Study [Galesloot et al. 2017], a population-based survey set up by the Radboudumc in 2000 in which randomly selected inhabitants of the municipality of Nijmegen received an invitation to fill out a postal questionnaire and to donate blood samples. The Arnhem-Nijmegen Regional Committee on Research Involving Human Subjects approved the AGORA project, the Nijmegen Biomedical Study, and the current study. The board of directors of the Isala clinics and UMCG approved implementation of AGORA in these centers. All participants and/or their parents gave written informed consent for participation in the study.

## German cohort

The German sample was recruited through CaRE for LUTO (Cause and Risk Evaluation for LUTO) study. Written informed consent was obtained from all subjects or their proxies, in case of minors. Patients with PUV were recruited in centers specialized for prenatal diagnostics, pediatric nephrology and or pediatric urology in Bonn, Marburg, and Regensburg. Demographic information was collected from patients upon inclusion. This study was approved by University of Bonn Institutional Ethics Committee (Lfd. Nr. 079/19). The control cohort has been used previously and information about general characteristics can be found elsewhere [Schmermund et al 2002]. As PUV is a male limited phenotype only male controls were used.

## Polish cohort

The sample of patients with Polish ancestry was sampled in collaboration of CaRE for LUTO with the PolTubeReg (Polish registry for tubular kidney disease). Written informed consent was obtained from all subjects or their proxies, in case of minors. Patients with diagnosed PUV were recruited upon routine follow up examinations in multiple centers specialized for pediatric nephrology all over Poland. Demographic information was collected from patients upon inclusion. This study was approved by each participating center's Institutional Ethics Committee and was conducted according to Declaration of Helsinki principles. The ethnical matched male control group was sampled, after informed consent from as blood donors in Lodz, Poland.

# References

Galesloot TE, Vermeulen SH, Swinkels DW, de Vegt F, Franke B, den Heijer M, de Graaf J, Verbeek ALM, Kiemeney LALM. Cohort Profile: The Nijmegen Biomedical Study (NBS). Int J Epidemiol. 2017;46:1099-1100.

Manders P, Lutomski JE, Smit C, Swinkels DW, Zielhuis GA. The Radboud Biobank: A central facility for disease-based biobanks to optimise use and distribution of biomaterial for scientific research in the Radboud university medical center, Nijmegen. Open J Bioresour 2018;5:2.

Schmermund A, Möhlenkamp S, Stang A, Grönemeyer D, Seibel R, Hirche H, Mann K, Siffert W, Lauterbach K, Siegrist J, Jöckel KH, Erbel R. Assessment of clinically silent atherosclerotic disease and established and novel risk factors for predicting myocardial infarction and cardiac death in healthy middle-aged subjects: rationale and design of the Heinz Nixdorf RECALL Study. Risk Factors, Evaluation of Coronary Calcium and Lifestyle. Am Heart J. 2002;144:212-8.

van Rooij IA, van der Zanden LF, Bongers EM, Renkema KY, Wijers CH, Thonissen M, Dokter EM, Marcelis CL, de Blaauw I, Wijnen MH, Hoogerbrugge PM, Bokkerink JP, Schreuder MF, Koster-Kamphuis L, Cornelissen EA, Kapusta L, van Heijst AF, Liem KD, de Gier RP, Kuijpers-Jagtman AM, Admiraal RJ, Bergé SJ, van der Biezen JJ, Verdonck A, Vander Poorten V, Hens G, Roosenboom J, Lilien MR, de Jong TP, Broens P, Wijnen R, Brooks A, Franke B, Brunner HG, Carels CE, Knoers NV, Feitz WF, Roeleveld N. AGORA, a data- and biobank for birth defects and childhood cancer. Birth Defects Res A Clin Mol Teratol. 2016;106:675-84.

# Supplementary Figures

***
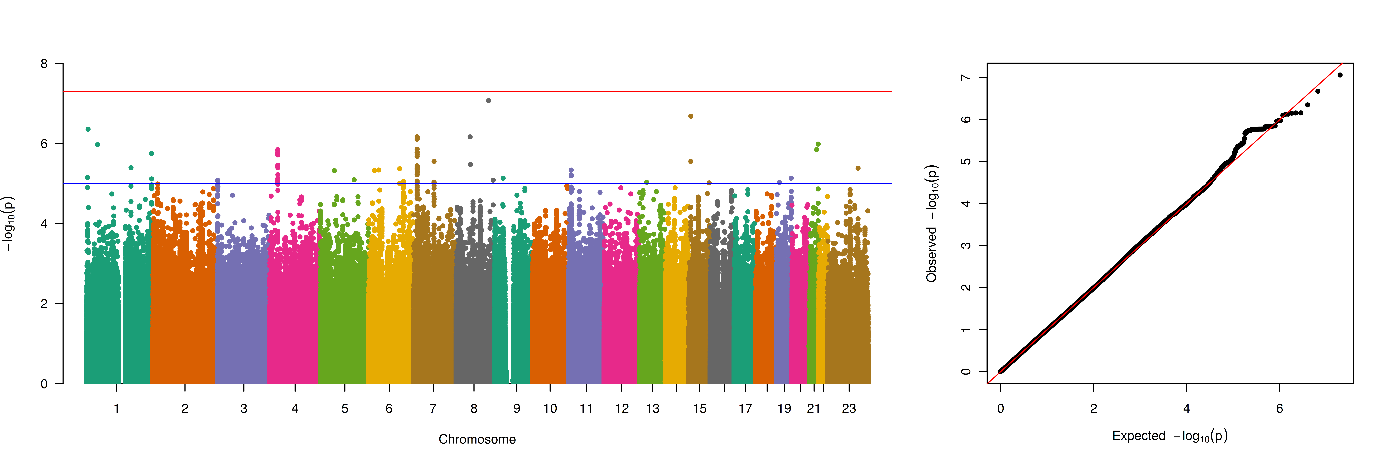
***

**Supplementary Figure 1.** Manhattan and quantile-quantile (QQ)-plot of pooled genome-wide association study results for the 9,885,387 variants that were genotyped and passed quality control in the Dutch Omni cohort of 402 PUV patients and 2,213 controls. The blue line indicates the threshold for suggestive genome-wide significance (p-value of 1×10^-5^) and genomic inflation factor λ=1.00.

*
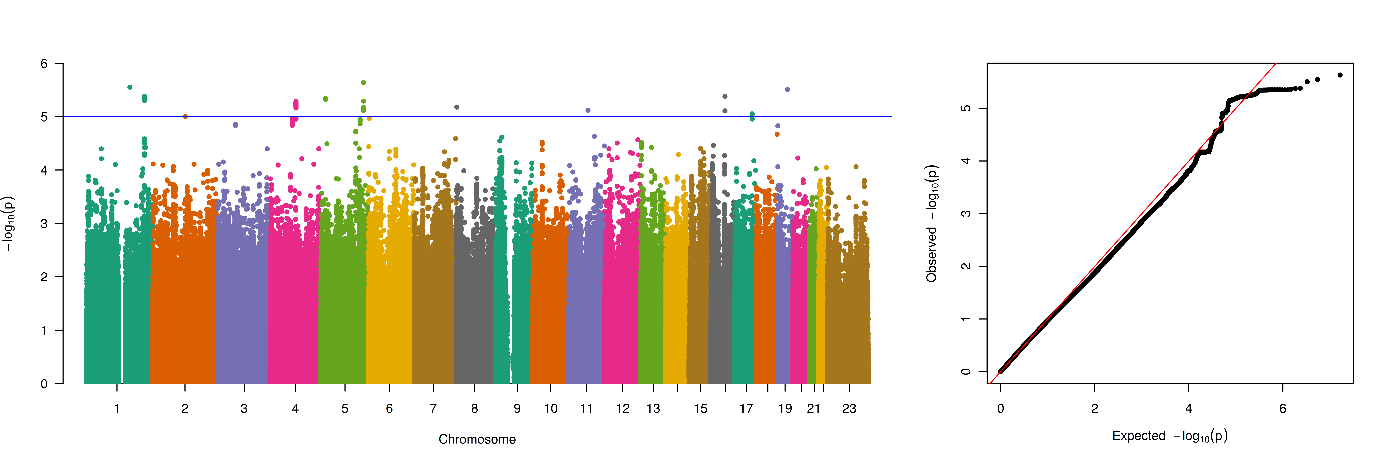
*

**Supplementary Figure 2.** Manhattan and quantile-quantile (QQ)-plot of pooled genome-wide association study results for the 8,200,150 variants that were genotyped and passed quality control in the Dutch GSA cohort of 58 PUV patients and 314 controls. The blue line indicates the threshold for suggestive genome-wide significance (p-value of 1×10^-5^) and genomic inflation factor λ=1.00.

.***
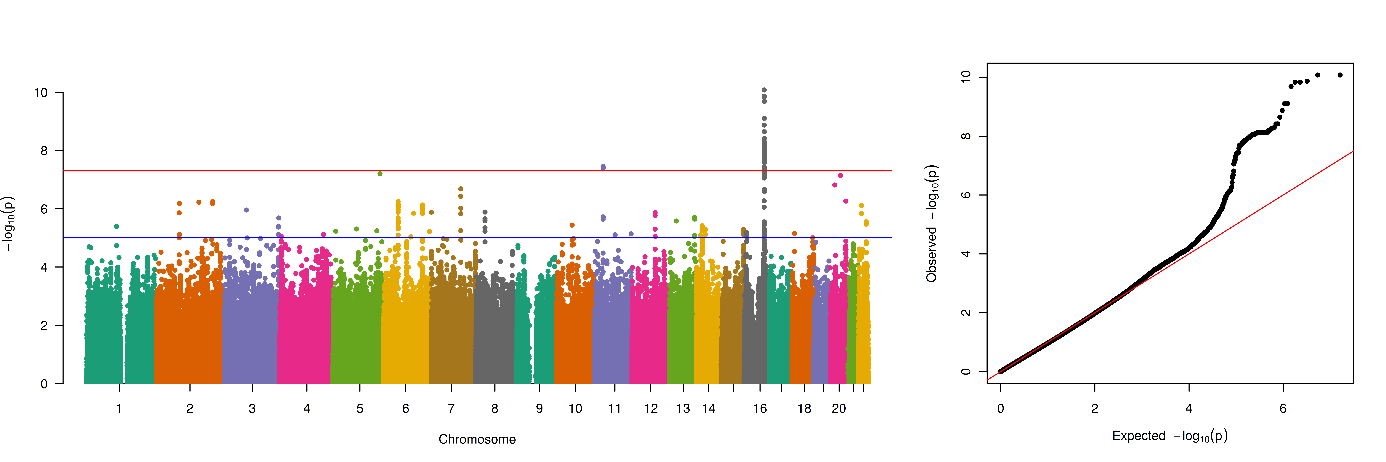
***

**Supplementary Figure 3.** Manhattan and quantile-quantile (QQ)-plot of pooled genome-wide association study results for the 8,088,657 variants that were genotyped and passed quality control in the German cohort of 104 PUV patients and 2,070 controls. The blue line indicates the threshold for suggestive genome-wide significance (p-value of 1×10^-5^) and genomic inflation factor λ=1.01.

***
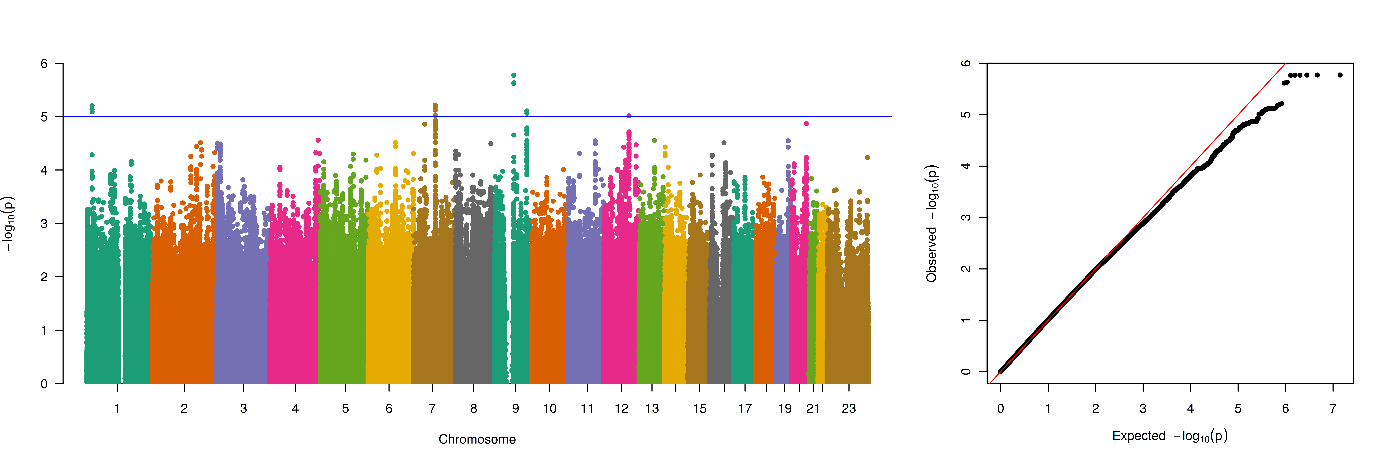
***

**Supplementary Figure 4.** Manhattan and quantile-quantile (QQ)-plot of pooled genome-wide association study results for the 7,018,455 variants that were genotyped and passed quality control in the Polish cohort of 192 PUV patients and 226 controls. The blue line indicates the threshold for suggestive genome-wide significance (p-value of 1×10^-5^) and genomic inflation factor λ=1.03.

# Supplementary Table legends

**Supplementary Table 1.** Association results in the 4 cohorts of PUV patients and controls and the meta-analysis for the 78 variants in the locus on chromosome 16 that yielded genome-wide significant results (P< 5×10^-8^) in the German cohort of 104 PUV patients and 2,070 controls.

**Supplementary Table 2.** Meta-analysis results for the 33 variants that showed suggestive significance (P< 1×10^-5^) in the meta-analysis of variants that were genotyped and passed quality control in all 4 cohorts of PUV patients and controls.
